# Gaming the unknown: learning to differentiate and respond to uncertainty through a serious game

**DOI:** 10.1038/s44168-025-00331-5

**Published:** 2026-01-10

**Authors:** Wout Jan-Willem Sommerauer, Bregje van der Bolt, Saskia Werners, Wouter Julius Smolenaars, Marlies van Ree, Fulco Ludwig

**Affiliations:** 1 Wageningen University and Research, Earth Systems and Global Change, Wageningen, Netherlands; 2 United Nations University, Institute for Environment and Human Security (UNU-EHS), Bonn, Germany; 3 Wageningen Environmental Research, Team Regionale Ontwikkeling en Ruimtegebruik, Wageningen, Netherlands; 4 International Water Management Institute (IWMI), Colombo, Sri Lanka

**Keywords:** Environmental social sciences, Psychology, Psychology

## Abstract

Effective climate adaptation planning requires understanding various sources and types of uncertainty. We investigated how a serious game can improve participants’ understanding of uncertainty in adaptation decision-making. Using mixed-methods with pre-post surveys and debriefing sessions, we engaged 55 university students in an adaptation pathways game simulating regional planning challenges over five decades with disruptive events. Results showed participants shifted from appreciating uncertainty as either environmental or institutional, to incorporating political shifts, institutional dynamics, and implementation challenges. Participants preferred flexible strategies over scenario optimization, with political disruptions generating stronger reactions than environmental shocks. Cognitive learning about uncertainty types facilitated normative learning effects, shifting strategic preferences. Participants recognizing diverse uncertainty dimensions became less confident in controlling outcomes, favouring more flexible and resilient strategies. Our findings, with contextual limitations, indicate serious games could help stakeholders develop an appreciation for adaptation approaches that maintain multiple options in parallel over single-pathway sequential solutions under uncertainty.

## Introduction

Climate change, characterized by rising temperatures and increasingly extreme weather events, poses significant risks to communities, ecosystems, and infrastructure globally. Addressing these challenges requires navigating uncertainty in dynamic and interconnected land-water systems under conditions of non-linear change, where the impacts are often difficult to predict or evaluate^1^. As a result, effective climate adaptation strategies are needed. Nonetheless, decision-making in this context is complicated by deep uncertainties regarding future climate conditions, population dynamics, and technological development^2^. Traditional planning approaches that rely on single or likely future scenarios could be complemented with a greater appreciation of ontological uncertainty, particularly when adaptation must move beyond incremental adjustments and towards transformative change. Which is relevant in a world likely to exceed 1.5 °C of warming, where both immediate actions to sustain existing systems and long-term strategies to create new ones are necessary^3–5^. Strategic choices around resource allocation and policy implementation must therefore be made under conditions of uncertainty^6,7^, while long-term climate projections remain limited in their precision and reliability^8^. Decision-makers are thus challenged to balance near-term decisions with long-term objectives in socio-ecological systems shaped by uncertain and evolving drivers^9^.

Pathway development has emerged as a response to this challenge, offering an approach to adaptation planning under uncertainty. By exploring multiple future scenarios and promoting collaborative learning, adaptation pathways facilitate more robust and flexible planning processes^10^. Adaptation pathways should not be treated merely as forecasting tools, but as instruments of change drawing on lessons from the past and our current understanding of how the world works and may change^11^. The effectiveness of using adaptation pathways is dependent upon how participants engage with uncertainty throughout the planning process^12^. Here, we categorize uncertainty based on Dewulf and Biesbroek^13^ framework, which distinguishes between epistemic uncertainty (knowledge gaps), ontological uncertainty (inherent unpredictability), and ambiguity (divergent interpretations). A challenge emerges from stakeholders lacking the intent and/or skills to differentiate between these different uncertainty types^14^. For example, ontological uncertainty is often treated as epistemic uncertainty or relegated to the background in favour of more ‘manageable’ pathways based on more familiar scenarios’^14,15^. The preference for more manageable pathways comes from a variety of factors such as uncertainty aversion being more important than risk aversion, aiming to keep specific options open or to avoid change^16^. Furthermore, the different interpretations of irreducible uncertainty make it difficult for planners to incorporate this form of uncertainty in long-term planning^14^. These tendencies introduce systematic biases that constrain adaptive flexibility and limit the potential of adaptation planning. Improving stakeholders’ understanding of ontological uncertainty can therefore support existing adaptation planning.

To address this limitation, we explore the use of a serious game to foster a deeper, more experiential understanding of (ontological) uncertainty in climate adaptation. Serious games are likely to be beneficial as they support communication, stakeholder engagement, and collaborative problem-solving in complex socio-environmental contexts, aligning with the outlined challenges in mainstreaming diverse uncertainty types^17,18^. When applied successfully, they provide structured, immersive experiences that simulate decision-making under complex and uncertain conditions. They have been employed across a wide range of sectors, from education to healthcare to public policy, as tools to enhance learning, critical thinking, and participatory engagement^19,20^. In the context of adaptation pathways, serious games offer a platform for scenario exploration, consequence evaluation, and the development of adaptive capacity^21^. Drawing on the principle of deliberate practice, these games involve goal-oriented tasks, real-time feedback, and iterative decision cycles that challenge participants to grapple with uncertainty^22–24^.

In this study, we employ a serious game that simulates a 50-year adaptation planning process, divided into five 10-year decision cycles. Participants take on the role of decision-makers tasked with implementing climate adaptation policies in pursuit of a resilient future vision, while responding to unpredictable events that influence the perceived legitimacy and effectiveness of their decisions^24,25^. These choices result in ex-post pathways that reflect the choices on adaptation made while playing the game. The game environment is based on the sandy soil regions in the eastern part of the Netherlands. These areas are particularly vulnerable to climate impacts due to the low water-holding capacity of the soils^26^. This vulnerability is further exacerbated by reliance on local water resources^27–29^. To study the effect of the serious game on a changing understanding of diverse uncertainty types, our research question is as follows: To what extent can a serious game that simulates uncertainty stimulate stakeholders’ learning about uncertainty in climate adaptation planning?

To answer this question, we assess changes in stakeholder understanding through surveys, before, during and after the serious game based on the survey design by Van Pelt. et al.^30^. To evaluate this change in understanding of uncertainty, we combine the uncertainty framework by Dewulf and Biesbroek^13^ with Baird. et al.^31^ distinction between cognitive learning: *changes in knowledge and understanding*, and normative learning: *shifts in values, problem frames, and decision logics*. We apply these frameworks in a particular order. First, we coded the data from the before and after game surveys using the uncertainty framework, after which we inductively identify themes in the coded material. We then apply these themes to the dataset (including in-game surveys and after-game discussion), and finally, we apply the learning framework to infer learning effects.

## Methods

### Research design overview

**Fig. 1:**
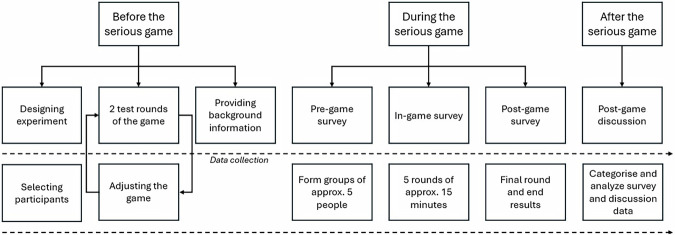
steps in the serious game experimental set-up.

This study employed a mixed-methods approach to investigate how a serious game can influence participants’ understanding of uncertainty in climate adaptation decision-making. Following Van Pelt et al.^30^, we used a single-group pre-post design with university students. The study involved 55 participants divided into 11 groups using a within-subject design, limiting the strain on participants and researchers. By utilizing this approach, we directly compared pre- and post-game views on uncertainty. The relatively homogenous group of MSc students in either climate sciences or international land and water management at Wageningen University shared a foundational knowledge of climate change, adaptation planning, and basic systems thinking. A summarized overview of the steps and process can be found in Fig. 1.

### The serious game

The pathways game used in this experiment was developed and extensively tested with stakeholders as a tool for engaging with decision-making under uncertainty, a core component of pathways methodology^9,24,25^. The game focused on navigating uncertain futures through strategic decision-making. Players assumed the role of decision-makers working to achieve a climate-resilient vision over a 50-year timeframe, divided into five 10-year rounds that culminate in strategically designed pathways^25^. Based on the Netherlands 2120 vision by Baptist. et al^32^. and tailored specifically to sandy soil landscapes, the game leveraged the diverse climate challenges and land use characteristics of these environments to simulate various climate change impacts and institutional uncertainties. This approach allowed for the introduction of multiple uncertainty dimensions within a complex land-water system, making it well-suited for experimental research and building on previous successful adaptation gaming methodologies^23,24,33^.

Prior to the game, students received a one-pager outlining the goals of the game, some background information on the case-study area (see *supplementary information (*SI5)), and the measure sheets to be used during the game. This information was repeated during the introduction of the game, which was further elaborated upon by stressing important game factors such as the role of the participants: civil servant, the aim; responding to future change, and urgency; the game is fast-paced with about 1,5 hours of actual gameplay. Additionally, we introduced our data collection, introducing what data we collect and informing participants of the ethical permissions given beforehand. Notably, no information was given on the subject of the study, barring the information participants could deduce from the actual data collection (i.e., the survey questions). Furthermore, the group of participants, as first-year master's students, had prior knowledge of climate change issues and adaptation through coursework. The period in which the experiment was planned saw the students having to implement adaptation plans based on data analysis. To facilitate this, students were introduced to various forms of uncertainty in relation to models, SSP’s and scenario-based thinking prior to the described experiment.

**Fig. 2:**
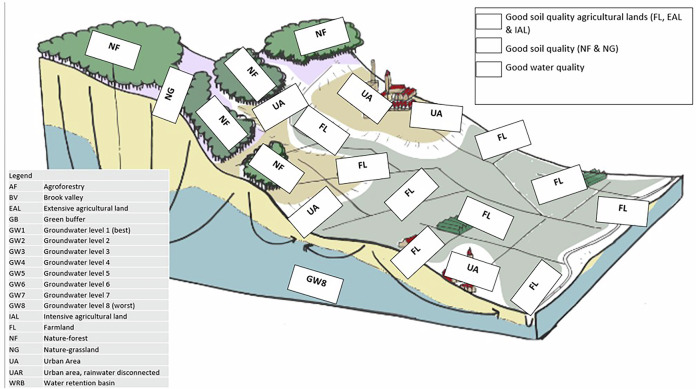
Initial set-up of the serious game based on the NL2120 vision by Baptist et al^32^. The map simulated an archetypical sandy soil landscape of the eastern Netherlands, with forest on the more elevated and drier areas, housing on the flanks and agriculture in the wetter brook valley. The white squares indicated land-use types, the groundwater table and potential soil or water quality improvement options. The map was originally altered for the serious game by Prins^24^.

The game structure consisted of five rounds, each simulating 10 years of change, where participants received limited resources (time relative to available measures) to implement interventions that transform a current situation map towards a (climate-resilient) vision map (see Fig. 2, for more details, SI3). Participants worked in groups of approximately five, and they collaborated to plan and design adaptive strategies over the course of 1.5 hours of gameplay. The vision map is introduced at the start of the game by describing common characteristics and climatic challenges on the sandy soils. Starting in round two and occurring in all subsequent rounds, participants encountered disruptive events that affected scoring, current conditions, or vision objectives, with bonus or penalty points awarded based on their responses to these challenges. Though the events could be considered feasible given the context, information on the timing and content was not provided during the introduction. The events introduced in this case study include: a flood event in round two that prioritizes water retention and shifts housing development patterns; a worldwide food crisis in round three that pressures policymakers to increase food production for relief efforts; a populist electoral victory in round four that elevates landscape preservation over nature restoration; and rapid protein transition in round five that reduces agricultural land-use demand due to declining animal protein consumption. These events were selected to introduce different forms of uncertainty by connecting a variety of potential impacts related to both the events themselves and the immediate response.

The game incorporated 4 unexpected events that impact the legitimacy and effectiveness of players’ choices. These events, combined with the vision map, introduce different ways uncertainty can manifest, allowing participants to engage with uncertainty in a controlled environment^21^. This design enabled participants to experience the implications of different decision strategies under uncertain conditions^34^ and to navigate these uncertainties by responding to unexpected events. Aligning with the game mechanics, we introduce uncertainty through the unexpected events, which are not only related to limitations in our understanding of the climate system, but also to the unpredictability of how society responds to climate change. This framing engaged participants with uncertainty at multiple decision-making levels. Uncertainty was thus expressed not only through the impacts of (extreme) climatic events but also via events simulating unpredictable societal change, such as shifting public preferences and politically or ideologically driven responses to global challenges.

The effectiveness of serious games in addressing uncertainty can be evaluated by comparing players’ uncertainty perceptions before and after gameplay, analysing the resulting learning effects. Van Pelt et al.^30^ emphasized experience’s role in shaping decision-making behaviour, suggesting that using relatively inexperienced students as participants might yield stronger observable learning effects. The framework for understanding these learning effects, as described by Baird et al.^31^, encompasses cognitive, normative, and relational learning. This study primarily focused on cognitive learning (understanding uncertainty) and normative learning (paradigm shifts), acknowledging their potential interconnection, as cognitive learning could facilitate normative learning. While relational learning remains important, it falls outside this study’s scope, as relevant adaptation decision-making tools such as the adaptation pathways approach inherently facilitate stakeholder interaction^35^.

### Data collection

The survey before the start of the game evaluated participants’ initial understanding of climate change uncertainty, including their knowledge of uncertainty sources in climate adaptation, decision-making approaches under uncertain conditions, and perceptions of adaptation planning. During gameplay, a brief survey captured decision factors for selected measures, how strategy evolved across rounds, and experienced certainty on decision making. Following the game, the survey mirrored the initial survey while prompting reflection on participants’ changed understanding, game experience, learning outcomes, and potentially changed perspectives on uncertainty management, in line with earlier work by Van Pelt. et al.^30^. This final survey particularly emphasized insights gained on handling uncertainty in adaptation planning (more details in SI1). The surveys before and after the game were conducted individually and the in-game surveys were distributed and completed on a group level.

Building on recommendations from Lawrence and Haasnoot^12^ and Blackett et al.^36^, we implemented a structured 30-minute debriefing process where the grouped participants discussed their game experience. In this debriefing, we stimulated a discussion on key insights, changed understanding of uncertainty, gameplay examples, and reflections on decision-making by introducing questions on uncertainty perception, specific revelatory moments, collective approaches to uncertainty, practical applications to climate adaptation planning, and potential future applications. For data collection from the debriefing session, we employed multiple documentation methods, including audio recording of the entire session, structured note-taking using templates based on the debrief questions, and facilitator documentation of notable quotes and observations.

### Analysis

All open-response coding was conducted by the first author using a structured codebook based on Dewulf and Biesbroek^13^ uncertainty framework. To enhance coding consistency and transparency, we established explicit decision rules with anchor quotes distinguishing between uncertainty types (more detail in SI4):

- Epistemic uncertainty: Statements framing uncertainty as knowledge gaps that could potentially be reduced through additional information (e.g., “not having full understanding of the climate system”).
- Ontological uncertainty: Statements emphasizing inherent unpredictability regardless of knowledge improvements (e.g., “you can’t predict elections, human behaviour”).
- Ambiguity: Statements focusing on divergent interpretations or context-dependent effectiveness (e.g., “social acceptance” of measures).

For the object of uncertainty, we distinguished:

- Substantive: Uncertainty about outcomes, impacts, or physical changes.
- Strategic: Uncertainty about actor behaviour, responses, or decision-making.
- Institutional: Uncertainty about governance structures, policy continuity, or organizational alignment.

We acknowledge that single-coder approaches introduce potential for interpretive bias. To mitigate this, we: (1) applied codes systematically across all responses before beginning analysis, (2) triangulated described outcomes with statements from debriefing sessions where participants spontaneously raised similar concepts if possible, and (3) focused analysis on large-magnitude shifts where coder interpretation would be less likely to alter overall patterns. Readers should interpret our coding as one plausible and systematic interpretation of participants' expressed understanding rather than definitive categorization.

To analyse both the survey responses and debriefing data, we developed a codebook informed by the uncertainty framework of Dewulf and Biesbroek^13^, this framework was used to code the survey data with the purpose of enabling more inductive exploration of themes. Additionally, it allowed for quantitative analysis of the object and nature of uncertainty, before and after the game. The nature of uncertainty included epistemic (knowledge-based), ontological (inherent unpredictability), and ambiguity-related (interpretive) forms. The object of uncertainty was categorized as substantive (related to outcomes), strategic (related to actor behaviour), or institutional (related to governance or rules). Given the game’s explicit focus on ontological uncertainty, emphasizing the unpredictability of responses to climate change, we expected only limited engagement with ambiguity-related uncertainty. The inductively found themes are subsequently applied to the entire dataset to ensure no relevant data is missed.

To move from the identified themes to cognitive and normative learning effects, the themes provided a starting point to compare participants’ understanding before and after the game (more detail in SI2). The learning patterns aimed to capture changes in participants’ thinking. Cognitive learning was defined as the acquisition of new knowledge, improved systems understanding, and awareness of decision-making implications. Normative learning was used to capture shifts in values, changes in strategic preferences, and broader paradigm changes in how participants conceptualize adaptation planning^31^. We supplemented the qualitative analysis with a quantitative comparison of before, during and after game survey responses, to extend the analysis of the found themes and learning patterns. This allowed us to assess changes in participants’ understanding of uncertainty over the course of the game. We compared rankings and perceptions of uncertainty before and after gameplay, presenting these findings alongside the qualitative data. This mixed-methods approach enabled a more nuanced understanding of how the game influenced participants’ learning and engagement with uncertainty in the context of climate adaptation planning.

## Results

The results describe participants’ understanding of uncertainty throughout the serious game experience, revealing changes from initial understanding to post-game perspectives. We start by examining how participants’ perceptions of uncertainty shifted between before and after the game, noting their evolving conceptualization of different uncertainty types. We then describe how their responses to uncertain conditions developed during gameplay, as teams adapted their strategies across the five rounds. Finally, we analyse the cognitive and normative learning patterns that emerged, examining how participants’ understanding and decision-making approaches were influenced by the game experience.

### Participants perceptions of uncertainty

Before gameplay, participants demonstrated a relatively compartmentalized view of uncertainty, primarily anchored in climate scenarios and their direct physical implications. The pre-game view was characterized by a focus on direct external drivers like climate variability, flooding, and drought (16 participants), with participants typically viewing uncertainty as something to be anticipated and managed (14 participants) through conventional top-down planning approaches (12 participants) that showed limited consideration of systemic relationships. The post-game discussion revealed how this climate-centric anticipation had shaped initial gameplay strategies. When asked what new insights participants gained about uncertainty, one acknowledged*: “[we] didn’t think about the political or the food crisis, we were really anticipating droughts”*. The discussion moderator’s observation that multiple participants shared similar expectations *“ I remember a group in that corner also saying something like, oh, the next thing must be a drought”* highlighted the focus on biophysical climate impacts. This emphasis on direct climate impacts left participants surprised by the social and political disruptions that would emerge during gameplay.

After playing the game, the survey data indicated a shift towards acknowledging complexity, including systemic drivers and institutional factors. Post-game, strategic (35%) and substantive (33%) uncertainty became approximately equivalent in prominence, representing a shift from the pre-game dominance of substantive uncertainty (44%) (see Table 1). Additionally, institutional uncertainty grew from 23% to 32% of mentions, reflecting implementation challenges and governance dynamics that became apparent during the game. Analysing the quotes from the open answers, epistemic uncertainty (knowable unknowns) consistently represented a significant part of the described uncertainty, increasing slightly from 33% to 34% of all uncertainty references. Ontological uncertainty (inherent unpredictability) remained the largest category growing from 44% to 48%, while ambiguity (unclear problem definitions) decreased proportionally from 23% to 18%. This pattern suggests participants increasingly recognized limits to predictability while becoming more conscious about problem boundaries. As one participant reflected: *“We must adjust and prepare for the uncertainty that comes from social attitudes and the ruling government”*. With another participants mentioning: *“using measures that are adaptive can more easily be made fit with unexpected scenarios”*.

**Table 1:** Overview of all relative sizes (as a percentage of the total no of answers) within the pre-game and post-game dataset of the coded objects and nature of uncertainty

Pre-game	*Epistemic*	*Ontological*	*Ambiguity*	*Total*
*Substantive*	15%	19%	10%	44%
*Strategic*	10%	15%	8%	33%
*Institutional*	8%	10%	5%	23%
*Total*	33%	44%	23%	100%
**Post-game**	*Epistemic*	*Ontological*	*Ambiguity*	*Total*
*Substantive*	12%	16%	5%	33%
*Strategic*	10%	18%	8%	35%
*Institutional*	13%	14%	5%	32%
*Total*	34%	48%	18%	100%

Analysis of coded uncertainty mentions revealed significant increases in both ontological (35 to 75 mentions, χ² = 14.55 df = 1, *P* < 0.001, φ = 0.36) and epistemic uncertainty recognition (26 to 53 mentions, χ²=9.23 df = 1, *P* = 0.002, φ = 0.34), suggesting participants identified more uncertainty overall rather than simply recategorizing existing concerns. The object of uncertainty also shifted: institutional uncertainty mentions increased most (18 to 50 mentions), followed by strategic uncertainty (26 to 55 mentions). Substantive uncertainty mentions increased moderately (35 to 51), though this did not reach significance (See SI6.1; Table SI6.1).

Participants developed awareness of system interconnections throughout the game. One participant during the post-game discussion noted that specific land-use types such as intensive agriculture are particularly vulnerable because *“there are too many factors that make it vulnerable like both socio-economic as well as environmental”*. This is indicative of how increased awareness of interconnectedness also informs participants of cascading effects of climate change impacts. In the post-game discussion, one of the participants explicitly stated players gained *“a more complete idea… of different types of uncertainties that can come”*. Where a different participant described how their group initially focused only on droughts and floods but was *“most surprised”* by the food crisis, recognizing diverse uncertainties beyond direct climate impacts. The serious game effectively highlighted multiple dimensions of uncertainty that participants had not previously considered in adaptation planning. One participant highlighted the impact of unpredictable events*: “The event cards really influenced uncertainty. It changed the way we selected measures because we were trying to anticipate for scenarios from the event cards but of course we had no idea what it could be”*. Not all participants found the game equally effective. Approximately 11 participants (20%) reported the game did not substantially enhance their understanding of uncertainty (ratings ≤3/5). Analysis of these responses suggests heterogeneous reasons: some participants (n = 4) indicated they already possessed sophisticated understanding of uncertainty from prior coursework *(“It showed the complexity which I was already aware of”*), others (n = 3) found the game’s simplified mechanics did not capture real-world complexity *(“This game is an incomplete situation… so it didn’t change my understanding much on a fundamental level”*), and several (n = 4) expressed that insights were not revelatory *(“It broadens my vision but doesn’t deepen my understanding”*).

**Fig. 3:**
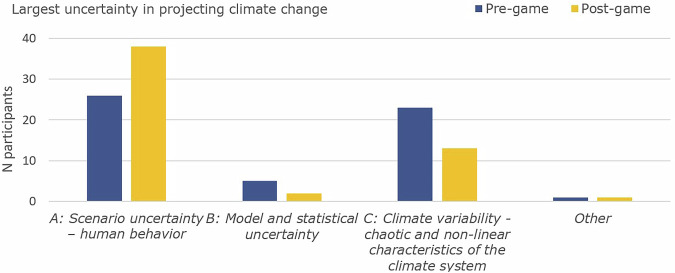
largest uncertainty in projecting climate change according to participants based on a multiple choice question before and after the game.

The political dimension emerged particularly strongly, with Speaker 1 finding political shifts impactful: *“That experience of the frustration [of political change] is very visceral”* and *“that’s actually happening now in the real world”*. Another participant contextualized this within their broader experience: *“when you’re involved with climate in general and adaptation measures, everywhere you look, you always get faced with uncertainty about this sort of build this”*. The political event (populist government) prompted a strong reaction, even though political events could often be considered reversible in contrast to some environmental changes due to climate change. The reactions to these events indicate that the game expanded participants’ understanding of uncertainty beyond environmental factors to include the social and political dimensions that can influence adaptation outcomes. Which was also evident in a slight shift towards naming human uncertainty as the largest uncertainty in project climate change (Fig. 3). The impact of this shift was evident when one participant expressed pessimism about the relationship between planning and politics in the post-game discussion: *“I feel even more insecure about adaptation planning now because once the populist show up, they’re [adaptation measures] being tossed out of the window… [even]If you plan in the perfect scenario, they don’t come out and then break it apart again as well”*. This represented a stark contrast from the pre-game survey quote that *“if there is a will in the political world, it will happen”*.

**Fig. 4:**
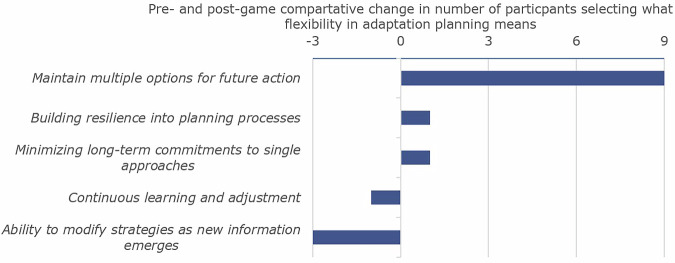
Change in perception on what flexibility means in adaptation planning comparing answers pre- and post-game. Calculated using the difference between the pre- and post-game answers to the multiple-choice question on what flexibility in adaptation planning means.

Nearly two-thirds of participants identified either alignment of organizations and governments (23) or practical implementation (15) as the most critical factor for effective adaptation pre-game. Most respondents post-game preferred adaptation measures that are adaptable and flexible (42) rather than tailored to specific scenarios (4), further supported by a shift in preference of 9 participants for maintaining multiple options post-game (see Fig. 4). This preference was supported by Speaker 4’s description of investing in “*soil quality and water quality*” as *“generally good”* regardless of future scenarios, showing incentive to find measures that provide long term flexibility. Arguing that these measures are unlikely to be challenged or otherwise impacted by the various events, and therefore keep other options that could respond to events open.

### Responding to Uncertainty

In-game survey results show a development in decision-making strategy throughout the game. Initially, most teams prioritized long-term vision, with 82% (9/11 groups) reporting no strategic impact in round 2, reflecting confidence in their ability to anticipate future conditions. However, environmental events and time constraints increasingly disrupted these strategies. By rounds 3-4, over half the teams (6/11 in round 3, 8/11 in round 4) reported strategy impacts demonstrating growing recognition of uncertainty (Table SI6. 4). Group certainty ratings showed a bell-shaped pattern across rounds. Teams reported moderate certainty in round 2 (M = 6.05, SD = 1.88), which increased in round 3 (M = 6.73, SD = 1.35), before comparatively declining in round 4 (M = 6.05, SD = 1.42) and round 5 (M = 6.10, SD = 1.73). While effect sizes comparing round 3 to other rounds were moderate (round 2–3: d = 0.42, round 3-4: d = 0.49, round 3-5: d = 0.44), differences did not reach conventional significance thresholds (all P > .14), likely due to limited statistical power with 11 groups (see Table. SI6. 3). Nevertheless, the pattern suggests participants initially gained confidence in their adaptive responses before implementation realities eroded certainty in later rounds. Looking at individual groups: 3 of 11 groups maintained high certainty, 4 groups showed declining confidence as the game progressed. Teams that focused on resilience and adaptability tended to remain certain, whereas those oriented toward specific outcomes were more vulnerable to change. These dynamics suggest that experienced preparedness for uncertainty could rely less on anticipating exact futures and more on keeping options open to preserve flexibility.

Gameplay observations revealed diverse responses to uncertainty. When the populist government event occurred in round 4, groups demonstrated contrasting reactions. Group 8 noted they *“were on the right road and need to align with the events card”* choosing to reverse previous nature-focused measures to align with the new political priorities. Their certainty actually increased to 8.5/10 in round 4, suggesting confidence in their adaptive capacity. In contrast, group 7 reported *“We sure got more insecure but still did things”* with their certainty dropping to 4/10, as they struggled to reconcile their long-term vision with political disruption. Strategic approaches also diverged in response to time constraints. Group 4 maintained adaptive capacity, noting they could still *“Get as much done as possible and aim for 80% match to final vision [and prepare for a] green transition”* demonstrating intentional maintenance of optionality. The flood event in round 2 elicited varied interpretations. Group 5 reported *“We anticipated the right thing and chose caution, got lucky that we focused on water safety”* framing their preparedness as strategic foresight. Meanwhile, Group 1 noted they were simply pursuing *“long term solution”* focused on *“Ground water level, [and to] realize [the] vision”*, citing they were unaffected because *“We did not do something that was affected by the flooding”*. This illustrates how external events generated different learning depending on groups’ strategic positioning.

The post-game discussion revealed development in strategic thinking across the gameplay experience. When asked what insights they gained, one participant reflected on the danger of overreacting to individual events: *“when we see one event, we might tend to change something, but actually in our experience we just left something the way it is and then the next event come about and actually that earned us some points… the turning point of the changes in the situation doesn’t necessarily mean that’s the new feature to stay, so there could still be more changes to come where our measures might even still work in the future”*, suggesting an appreciation of temporal complexity and potential chaotic nature of adaptation planning. Another participant described how their strategy emphasized maintaining broad options: *“we keep it flexible… …to increase resilience [by] investing [in] things that doesn’t matter what happens [are] just generally good”*. When asked about their approach if playing again, one participant who had initially adopted a *“go big or go home”* strategy acknowledged they would shift toward *“low regrets”* measures. Another emphasized they would implement *“more flexible options and there’s also just in general more uncertainty that I would think about”*. When the moderator probed whether feelings of gambling increased or decreased during gameplay, one participant captured the nuanced reality: *“I think it decreased, but even at the very end we were having a discussion… I think it decreases and it increases based on if you’re lucky”*. This indicates a recognition that uncertainty cannot simply be resolved through better planning, but must be actively navigated. Taking a more nihilistic perspective a group used chance in their decision-making, with one describing moments *“where we’re like, OK, choose a number, and then we’ll decide on if you guess the number correct. If you go for one or [another option]”*, illustrating how groups sometimes resorted to randomization when faced with irreducible uncertainty.

Reflecting on the game in the individual surveys, participants' perception of uncertainty changed throughout the serious game, revealing insights about implementation challenges that extend beyond theoretical planning. As one participant reflected, *“The more flexibility you have, the easier it is to adapt to changes in any situation, either geophysical or political”*. This highlights a key finding: adaptation involves not only planning under uncertainty but also navigating the uncertainties that emerge during implementation. Participants began to articulate a more sophisticated understanding of temporal dynamics in adaptation planning. When discussing lessons learned, one participant explained: *“the lesson that I find most important is basically to think in intervals instead of thinking from now to 50 years… if you were to think now what would happen in 50 years, what do I do to get there, you don’t think about the things that happen within those 10 year [steps] and that shorter term thinking within the long term, that’s something that I found very interesting”*. When asked by the moderator on whether this would change their approach *“if you were now the civil servant planning for that 15 years, would you change the decisions compared to before the game?”* the response was: *“I would [choose] more flexible options and there’s also just in general more uncertainty that I would think about”*. Ultimately, implementation realities eroded initial strategy adherence, with only a minority of teams maintaining their original approaches throughout the game.

When asked what they might use in future work or studies, one participant emphasized the importance of looking beyond immediate research boundaries: *“we don’t only look at inwards to what we’re doing, what we’re researching… it’s very important to really look outside to see how things are changing like weather wise or political wise… that external look is very important. That will give us maybe earlier signal on what we should focus on in the future”*. Another participant contextualized this within the broader challenge: *“there are things that you just cannot change by studying”*. This supports an evolution from the pre-game emphasis on anticipating specific climate scenarios, toward recognizing the need to monitor and respond to diverse external signals.

**Fig. 5:**
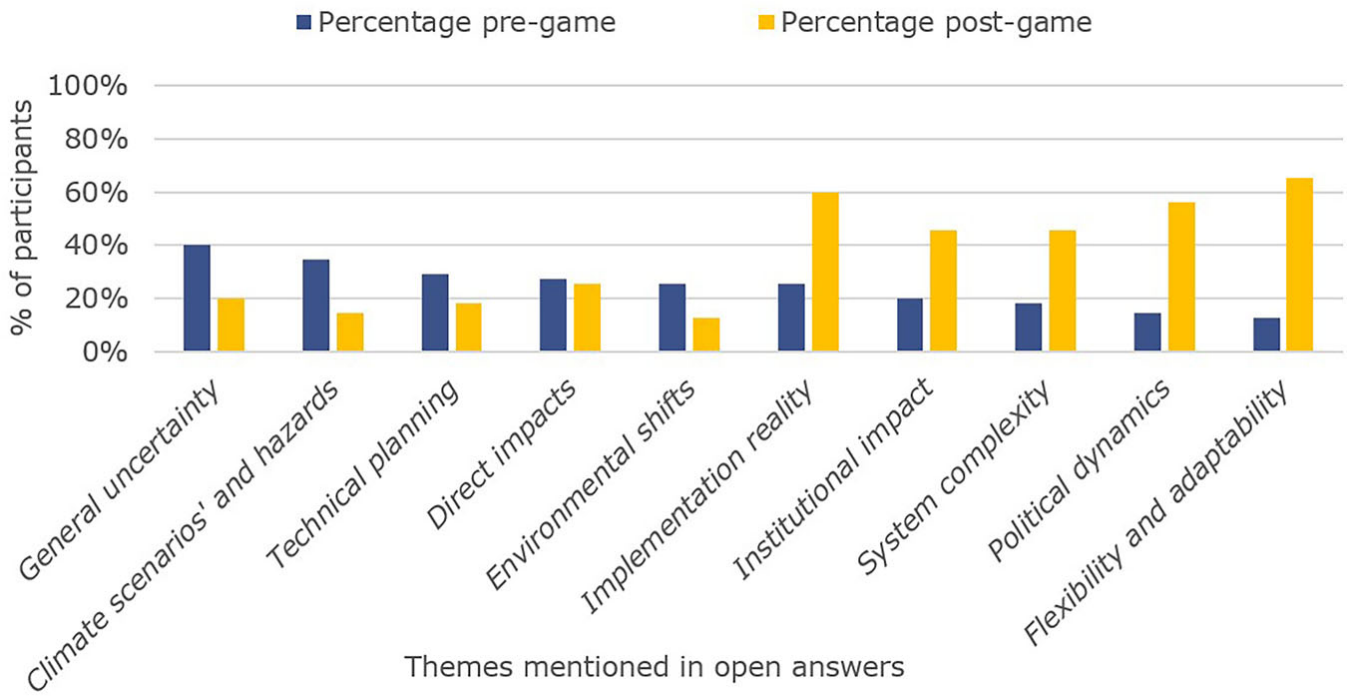
Thematic analysis of answers to open survey questions. The percentage represents the number of participants mentioning a theme in their open answers in comparison to the total group (*N* = 55).

Despite increasing time pressure and the difficulty of mid-game adaptations, participants reported a high level of appreciation for the serious game experience. On average, they rated the game as enjoyable (4.6/5, *N* = 54), valuable for enhancing their understanding of adaptation pathways (3.7/5, *N* = 54) and valuable for learning about different dimensions of uncertainty (3.8/5, *N* = 54). Changes in uncertainty perception based on the uncertainty framework showed several themes and subthemes. Participants referred to ontological uncertainty related to institutional object, uncertainty related to implementation (strategic), environmental uncertainty (substantive), and interpretation of adaptability and flexibility in response to these uncertainties (strategic) (see Fig. 5). Chi-square tests confirmed the significance of these thematic shifts. Recognition of political dynamics increased from 15% to 56% of participants (χ² = 13.56 df = 1, *P* < 0.001, φ = 0.5, medium to large effect), while mentions of flexibility and adaptation grew from 13% to 65% (χ² = 19.56 df = 1, *P* < .001, φ = 0.6, large effect). Institutional impact recognition increased from 20% to 45% (φ = 0.31), and system complexity awareness grew from 18% to 45% (φ = 0.34). Implementation reality mentions increased from 25% to 60% (φ = 0.37). Conversely, exclusive focus on climate scenarios decreased from 35% to 15% (φ = 0.29), suggesting participants broadened rather than merely shifted their understanding of uncertainty sources (Table. SI6. 2). Institutional uncertainty proved particularly disruptive (supported by the shift in recognition of political dynamics), as one participant reflected, *“My view on uncertainty is largely the same, although now with greater weight on the political aspects of uncertainty”*. This shift was also evident in survey data, where scenario uncertainty and human behaviour gained more attention than climate variability (see Fig. 3). Teams that acknowledged time constraints in later rounds were more likely to report strategic impacts, while those that built flexibility in early rounds were sometimes less affected. One participant remarked, *“I realised that you become more unsure of your strategy when your plan changes the first time, you get more scared of the uncertainty”*.

**Fig. 6:**
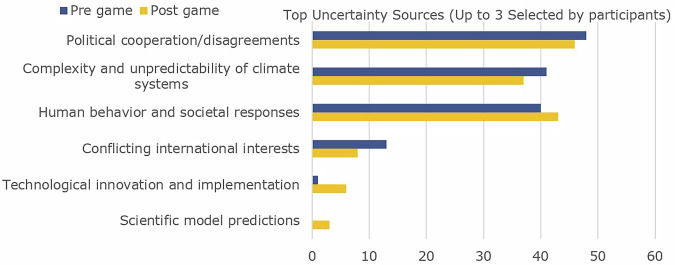
Most frequently selected sources of uncertainty by participants based on a closed question asking participants to select the 3 most important sources of uncertainty.

Survey data demonstrated that participants most frequently identified political cooperation (*n* = 48), human behaviour (*n* = 40), and climate system complexity (*n* = 41) as primary sources of uncertainty (Fig. 6). Despite the emergence of new thematic categories (Fig. 5), participants’ fundamental perceptions of uncertainty remained largely consistent between the pre- and post-game survey, in spite of shifting perspectives on political dynamics, implementation challenges, institutional impact, and system complexity. This consistency suggests that the serious game primarily challenged and refined existing uncertainty perceptions rather than fundamentally transforming participants’ conceptual frameworks or imposing deterministic perspectives through the selected event or survey design. The marginal reduction in emphasis on climate system complexity and unpredictability may, however, be attributable to the thematic shifts induced by specific in-game events and scenarios.

### Cognitive learning

**Fig. 7:**
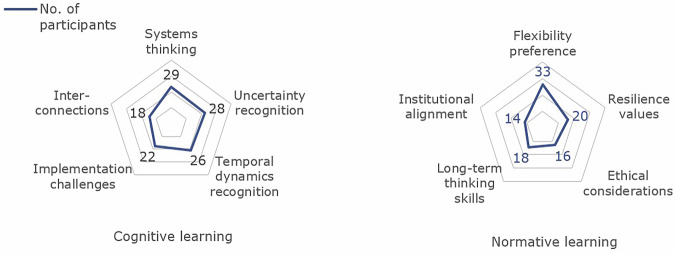
Combined observed learning themes from the study, encompassing both cognitive and normative dimensions. Cognitive learning is represented by five themes: Systems Thinking (understanding interdependencies and cascading effects), Uncertainty Recognition (acknowledging diverse forms of uncertainty beyond climate), Temporal Dynamics (recognizing adaptation as an iterative, time-sensitive process), Implementation Challenges (understanding the gap between planning and practice), and Interconnections (seeing how systems and stakeholders mutually influence outcomes). Normative learning is represented by five themes: Flexibility Preference (valuing adaptable over rigid approaches), Resilience Values (adopting resilience as a guiding principle), Ethical Considerations (emphasizing fairness and responsibility in adaptation decisions), Long-term Thinking (prioritizing generational over short-term perspectives), and Institutional Alignment (valuing coordination across organizations and governance levels).

The serious game stimulated cognitive learning effects (acquisition of new knowledge or structuring of existing knowledge^31^) across multiple dimensions, resulting in participants shifting from linear to systems thinking. This evolution is supported by an increase from 21/55 to 32/54 participants describing multiple forms of uncertainty by the end of the game, mentioning different forms of uncertainty that could impact adaptation efforts instead of focussing on a singular aspect such as physical or social change (see Fig. 7). Additionally, participants developed a more elaborate understanding of temporal dynamics, recognizing that adaptation is iterative in practice rather than exclusively endpoint-focused. The round-based structure to progress the game, comprising five sequential rounds, each representing a ten-year period with limited resources, fostered understanding of how decisions unfold over time, with participants recognizing limitations of planning exclusively for distant endpoints rather than adaptive pathways. This was reflected in an increase from 3/55 to 32/54 participants explicitly naming flexibility as a strategy (see Fig. 7).

Participants gained practical insights on implementation challenges, with uneasiness about the effectiveness of planning and implementation frequently mentioned. The gap between planning and implementation was observed across 22/54 participants (see Fig. 7). As one participant reflected: *“For example: we thought we were implementing effective measures, however in the end they were not as effective”*. Another noted: *“The fact that taking measures that in the following period were not desirable anymore gives me the feeling that often it is unfortunately not possible to plan long term because of uncertainty”*. Some participants demonstrated improved capacity to anticipate uncertain events, moving from reactive responses in early rounds to more strategic anticipation of potential disruptions (25/54 participants, Fig. 7). This was supported by the choice to maintain multiple options and avoiding early lock-in (42/54 participants), a perspective that aligns closely with the pathways approach and represents a cognitive advancement in adaptation planning capabilities. Flexibility was also acknowledged during the post-game discussion through concrete examples. When asked about changing their perspective on uncertainty, one participant noted: *“we can’t 100% [be] prepared, but it’s good to be aware of things that can happen”*. Another described how *“the game provided concrete examples of what can happen and what kind of surprises that can [occur]”*. The need for flexibility was primarily driven by the recognition of interconnections and systems thinking when confronted by adaptation planning (Fig. 7). Collectively, these findings suggest that participants developed a more nuanced understanding of adaptive planning, one that embraces uncertainty as unavoidable while building capacity for strategic flexibility and anticipatory decision-making.

### Normative learning

The serious game stimulated normative learning effects (learning related to viewpoints, paradigms and value shifts^31^), with a strong preference for flexible, low-regret strategies emerging post-game. As one participant stated: *“Adaptation measures should be flexible, because there can be a lot of change in the future you can’t prepare for”*. This represents an evolution from isolated goal-oriented thinking to valuing resilience and adaptability (20/54 participants, Fig. 7). This could also be observed in participants becoming comfortable acting despite uncertainty, moving from trying to predict futures to finding factors contributing to resilience (14/54 participants). This shift is illustrated by pre-game statements such as *“This uncertainty mostly refers to uncertainty that is for now impossible to reduce, so you can’t ever be absolutely certain”* compared to post-game reflections like *“Stay adaptive to tackle uncertainty for what happens after you planned for it”*. Participant perspectives on flexibility within adaptation planning also shifted, as illustrated by one participant whose view evolved from pre-game *“Not being absolutely sure about the factors involving climate adaption planning”* to post-game “*By being flexible it allows to adapt to changing conditions in climate but also in for example politics”*. Additionally, cognitive learning facilitated normative learning in a subset of participants: increased understanding of system complexity led to greater humility about control limits of the system (10/54 participants) and recognition of diverse uncertainty types prompted value shifts toward flexible approaches (33/54 participants). These interconnected learning processes ultimately influenced participants’ values about adaptation decision-making, with observable effects moving participants from certainty-seeking to uncertainty-embracing approaches that prioritize adaptive capacity over predictive control.

Debriefing responses revealed distinct strategies that had emerged. Some participants adopted precautionary approaches: *“We tried to anticipate for scenarios from the event cards but of course we had no idea what it could be”* demonstrating attempts to prepare for unknown unknowns. Others embraced opportunistic adaptation through iterative adjustment over long-term commitment. A third pattern emphasized robust baseline improvements, with one participant describing investing in *“soil quality and water quality”* as *“generally good”* regardless of future scenarios, indactive of low-regret strategies that maintain flexibility across multiple futures. These reflections, in conjunction with the survey results, provide triangulating evidence for systematic shifts observed in coded survey responses. Participants demonstrated increased willingness to make decisions under uncertainty by building flexibility into strategies, as experienced certainty about outcomes decreased. A subset of participants began incorporating ethical considerations alongside technical solutions (16/54 participants), showing deeper normative learning about value trade-offs inherent in adaptation planning (see Fig. 7). Furthermore, participants experienced increased skill in balancing immediate needs with long-term sustainability considerations (18/54 participants), as reflected in this observation: *“You don’t know what [events are] going to happen so using measures that are adaptable is always good. Also considering the long term, we skipped out on changing to [(intensive agriculture] in round 4 as we thought that this wouldn’t be sustainable in the long term, which turned out to be a good decision”*. Additionally, there was recognition of the role of institutional alignment and community response (14/54 participants) in successful adaptation strategies, representing a broader normative shift in how participants conceptualized effective climate governance (Fig. 7). However, these shifts should be understood as representing only a subset of all participants, highlighting that though learning is evident; the themes reflect specific clusters of learning within the group and context of this study.

## Discussion

The results of this study show how serious games can help participants engage with and respond to uncertainty in climate adaptation planning. The serious game applied in this case-study allowed participants to experience and engage with uncertainty, focusing on uncertainty arising from irreducible unpredictability (ontological uncertainty). Our findings highlight that institutional and governance uncertainties can be experienced as similarly disruptive to adaptation processes compared to climate system related uncertainty. Participants demonstrated both signs of cognitive learning (enhanced understanding of complexity, feedback loops, and uncertainty) and normative learning (shifts in values around knowledge, process, and time). These forms of learning reinforced each other and led participants to shift from predictive, risk-based planning toward more adaptive, systemic approaches. The game proved a valuable tool for developing adaptive capacity as players reframed uncertainty as something unavoidable rather than a problem to be solved. Serious games thus offer engaging, low-risk environments that support learning critical for navigating uncertain futures, an aspect currently considered challenging^9,12^. Further research is needed to see what parts of this learning persists^37,38^. These outcomes align with prior professional applications, where participants also found the game useful for unpacking uncertainty, engaging and informative for exploring adaptation strategies^24^.

Several methodological limitations should be considered when interpreting our findings. Our approach relied on researcher-coded self-reports without independent verification, and while we established coding rules, coding decisions reflect our interpretation of participants’ expressed understanding rather than definitive categorization, introducing potential for interpretive bias. The structured pre-game survey may have constrained participants’ initial responses through its multiple-choice format. This may make baseline understanding appear more compartmentalized than it actually was and limiting their ability to express nuanced perspectives. Additionally, post-game Likert items on flexibility and adaptation not listed in the pre-game survey may have introduced demand characteristics, as students could infer desirable responses after gameplay. Our research design mixed individual-level (pre/post surveys) and group-level (in-game surveys) data without hierarchical modelling. In response, we separated these analyses in our results, though future research could employ multilevel approaches to better capture individual trajectories within group contexts. Statistical power was also limited by sample size, particularly for group-level analyses (*N* = 11), and while effect sizes suggested meaningful changes in certainty across rounds (d = 0.42-0.49), these did not reach conventional significance thresholds. Individual-level analyses (N = 55&N = 54) provided sufficient power to detect large thematic shifts.

Our homogeneous sample of MSc students from one university limits generalizability to professional stakeholders. The use of MSc students proved effective for observing learning, as relatively inexperienced learners are often more receptive to shifting their problem frames^30^. The original game was co-designed with practitioners^24^. This is reflected in realistic decision-making dynamics through in-game events and aligning with probable career paths of the participants, enhancing relevance. Professional stakeholders potentially show more normative but less evident cognitive shifts given their established expertise. The single national planning context (Netherlands) and landscape type (sandy soils) further constrain transferability. However, several factors suggest these methodological limitations do not fully explain our findings: first, the magnitude of shifts was large (φ > 0.4 for key themes). Second, participants spontaneously raised similar themes in open debriefing discussions without leading questions with unprompted debriefing responses. Third, approximately 20% of participants did not report improved or significantly changed understanding of uncertainty, suggesting students felt comfortable expressing dissenting views rather than giving in to social desirability bias. Finally, behavioural indicators during gameplay (strategy disruptions, time allocation) supported self-reported shifts. Our findings, therefore, should be interpreted as indicative patterns of learning effects rather than definitive causal claims. Future research should employ more open-ended pre-game instruments and include parallel Likert items across timepoints to strengthen validity claims.

Our pre- and post-game survey results revealed that participants showed little change in identifying sources of uncertainty (e.g., climate system, political cooperation). Participants’ understanding of uncertainty shifted, with large effect sizes (φ = 0.5-0.6 for major thematic shifts) indicating substantive changes in how participants conceptualized uncertainty. This indicates that the game was more successful at strengthening participants’ *responses* to uncertainty rather than altering their foundational thinking about its *origins*. By designing gameplay around cognitive engagement with evolving and unexpected uncertainties, the game encouraged participants to question assumptions and reframe problems. This reinforces the value of adaptation approaches that focus on fostering response capacity, rather than attempting to predict specific uncertainties^13,34^. This kind of experiential learning, especially when tied to relevant planning challenges like the sandy-soil landscape context (based on the NL2120 vision by Baptist et al.^32^), can be a powerful and efficient way to prepare future professionals to navigate complex planning environments and support strategic thinking in adaptation pathways development.

Although the game fostered valuable learning effects, it remains unclear whether these insights translate into improved real-world planning. Awareness of uncertainty does not automatically lead to better decision-making^12,37^. Throughout and after the game, though, we did observe changes in (framing of) decision-making, demonstrating development by connecting different uncertainty types and future visions to find measures that contribute to long-term resilience. This transition, from a predictive mindset toward one emphasizing resilience and flexibility, complements existing scenario-based approaches by encouraging longer-term (50+ year) perspectives and expanding consideration beyond climatic or socio-economic factors to include political and other unpredictable influential developments. Embedding follow-up exercises, such as real or hypothetical pathway development tasks, could help evaluate whether players apply their learning to practical cases. Longitudinal studies could also track whether learning endures over time^39,40^. Integrating games into real planning processes, workshops, institutional training, or participatory governance, however, could likely constitute teaching tools into instruments of organizational learning and planning innovation based on the shifts observed in this study.

In conclusion, the game stimulated shifts in participants’ conceptual and strategic approaches to uncertainty, applying these lessons in practice, though it requires structured reflection and contextual support. The post-game debriefing was valuable in enabling participants to connect game experiences to real-world planning challenges. Participants emphasized political and institutional uncertainty as particularly disruptive, often more so than environmental shocks, a finding that aligns with earlier studies^15,41^. However, meaningful practice change requires more than awareness; it involves the application of new insights in organizational settings, persisting through existing planning cultures. Comparing how participants with varying levels of expertise experience serious games could help tailor them to different stakeholder needs, in particular shifts in cognitive versus normative learning, would enrich our understanding of how to foster transformative adaptation planning across diverse practitioner contexts^35,36^. Understanding how participant profiles influence both cognitive and normative learning outcomes will be consequential for scaling the application of serious games in stakeholder engagement and planning. Finally, engagement with ontological uncertainty dimensions, underrepresented in current pathways methods, could be enhanced by serious games that stimulate participants in the recognition, negotiation, and action to respond to uncertainty when planning climate adaptation in complex settings^9,38^. Letting games serve not just as educational tools, but as catalysts for more meaningful participation in adaptation pathways planning.

To conclude, this study investigated the extent to which a serious game simulating uncertainty can stimulate stakeholders’ learning about uncertainty in climate adaptation planning. Our findings demonstrate that serious games can facilitate learning, supporting both cognitive and normative learning processes that reshape how participants approach adaptation planning under uncertain conditions. Participants demonstrated cognitive learning through their evolution from appreciating uncertainty as either environmental or institutional, to a system perspective encompassing political, climatic, institutional, and social dimensions. The game also fostered normative learning with participants moving from seeking predictive control through scenario-specific solutions to embracing adaptive flexibility and strategies that maintain multiple options rather than optimizing for single predicted outcomes. Through repeated exposure to disruptive events, participants developed an appreciation for adaptation approaches that maintain multiple options in parallel rather than pursuing single-pathway sequential solutions. These learning effects directly address systematic biases that constrain adaptive flexibility in traditional planning frameworks by helping stakeholders move beyond treating ontological uncertainty as merely epistemic. Political disruptions particularly proved more experiential than environmental shocks, in part leading to value changes that prioritized resilience-oriented decision-making approaches over short-term certainty. The interconnected nature of these learning processes enhanced understanding of system complexity, which facilitated value shifts toward flexible, low-regret adaptation strategies. The translation of these insights into improved real-world planning practices requires further research, given the limitations of this study in relation to participants characteristics and the mixed significance of identified patterns. In spite of these limitations, serious games demonstrate potential to stimulate the learning necessary to move from predictive risk-based approaches towards adaptive systems-oriented planning that embraces uncertainty as inherent to complex socio-ecological systems.

## Supplementary information

Supplementary material

## Data Availability

The data that support the findings of this study are openly available through the 4tu data repository. 10.4121/fac2f69c-850e-4836-a76e-b38d65ebda17.
