# Supplementary material for: Gaming the unknown: learning to differentiate and respond to uncertainty through a serious game
Source: NPJ Clim Action. 2026 Jan 10;5(1):5. doi: 10.1038/s44168-025-00331-5 (PMC12790468; doi:10.1038/s44168-025-00331-5)
Supplement: Supplementary file 1 — Supplementary material [file 44168_2025_331_MOESM1_ESM.pdf]

SI1: Survey

Pre-game survey

Pre-game survey

Dear Participant,

You are invited to participate in a research study about serious games in climate adaptation planning. This study is conducted by Wageningen University & Research as part of research into innovative planning approaches.

Purpose

This research aims to evaluate the effectiveness of serious games for climate adaptation planning processes.

What You Will Do

Your participation will involve:
- Completing two surveys individually (before and after gameplay)
- Completing a set of group level surveys during the game
- Participating in a serious game session (approximately 2 hours)
- Joining a group discussion after the game which is audio recorded

Time Commitment

Total time commitment: approximately 2.5 hours

Data Protection and Privacy

All data will be collected anonymously
- Results will be analyzed at the group level
- Your responses cannot be traced back to you individually
- Data will be securely stored in password-protected files
- Only the research team will have access to the raw data

Voluntary Participation- Your participation is entirely voluntary
- You can withdraw at any time without consequences
- You can skip any questions you prefer not to answer

Use of Results

The results will be used for:
- Publications in (academic) journals
- Improving future serious games

Participants can withdraw at any time without any consequences for their course performance/evaluation"

Contact Information

For questions about this research, please contact: Wout Sommerauer

Email: Wout.sommerauer@wur.nl

Do you have questions on the ethical permission or remarks you want to share with someone independent from the study, please contact the Wageningen University and Research - Research Ethics committee (WUR-REC).

Email: REC@wur.nl

* Vereist

1. I have read and understood the information above and agree to participate in this research. *

○ Yes

2. What is your tracking number? *

[ ]

3. What do you consider to be the main sources of uncertainty in planning for climate change (select up to 3)?

Selecteer maximaal 3 opties.

- ☐ Limited (financial) resources
- ☐ Political cooperation/disagreements
- ☐ A lack of technological innovation and implementation
- ☐ Human behavior and societal responses
- ☐ Complexity and unpredictability of climate systems
- ☐ Conflicting international interests
- ☐ Andere

4. When developing climate change adaptation strategies, how important would you rate the following factors on a scale of 1-5 (1 = not important, 5 = highly important)

	1	2	3	4	5
Local variations in climate predictions and their accuracy	○	○	○	○	○
How climate solutions that work in theory might face practical difficulties when implemented	○	○	○	○	○
How communities might respond differently to climate change impacts and proposed solutions	○	○	○	○	○
The availability and distribution of funding for climate initiatives	○	○	○	○	○
How climate strategies might affect different sectors of the economy	○	○	○	○	○
How different organizations and governments align their climate actions	○	○	○	○	○

5. From the factors above, which do you believe is the **most** critical for effective **climate adaptation planning**?

- ◯ Scientific model predictions
- ◯ Practical implementation
- ◯ Community response
- ◯ Availability and distribution of funding
- ◯ Impact on different economic sectors
- ◯ Alignment of organization and governments

6. Please briefly explain your choice at question 5

7. How do you expect scientific research will impact our understanding of future climate uncertainties?

- ◯ Significantly decrease uncertainties
- ◯ Decrease uncertainties
- ◯ Maintain current uncertainty
- ◯ Slightly increase uncertainties
- ◯ Significantly increase uncertainty

8. When selecting climate adaptation measures as a spatial planner, I would prioritize measures that are: (select one)

- ◯ Robust against all climate change scenario's
- ◯ Effective against the most extreme climate change scenario
- ◯ Tailored to the most likely climate change scenario
- ◯ Adaptable and flexible across different scenarios
- ◯ Andere

9. At this moment, I believe the largest uncertainty in projecting climate change is: (Select one)

○ Scenario uncertainty - the unpredictability of human behaviour

○ Model and statistical uncertainty - limitation related to climate science and statistical methods

○ Climate variability - chaotic and non-linear characteristics of the climate system

○ Andere

10. Rate the importance of the following principles in adaptation planning:
(1 = Not Important, 5 = Extremely Important)

	1	2	3	4	5
Cost-effectiveness	○	○	○	○	○
Long-term sustainability	○	○	○	○	○
Community resilience	○	○	○	○	○
Ecosystem preservation	○	○	○	○	○
Equity and social justice	○	○	○	○	○

11. When making decisions about adaptation measures under uncertainty, I would: (Select one)

○ Develop multiple contingency plans

○ Prioritize **low** regret, flexible solutions

○ Focus on **no**-regret actions with immediate benefits

○ Invest in ongoing research and monitoring programs

○ Implement adaptive management strategies

○ Andere

10

11

12. Flexibility in adaptation planning means: (Select all that apply and feel free to add your own interpretation)

☐ Ability to modify strategies as new information emerges

☐ Maintain multiple options for future action

☐ Minimizing long-term commitments to single approaches (not putting all eggs in one long-term basket)

☐ Continuous learning and adjustment

☐ Building resilience into planning processes

☐ Andere

13. Rate your agreement with the following statement:
"Before implementing climate adaptation measures, I want absolute certainty about projected climate change impacts." (1 = strongly disagree, 5 = strongly agree)

1	2	3	4	5

14. Could you describe your understanding of **uncertainty** in climate adaptation planning?

12

13

Post game survey

Post-game survey

Dear Participant,

You are invited to participate in a research study about serious games in climate adaptation planning. This study is conducted by Wageningen University & Research as part of research into innovative planning approaches.

Purpose

This research aims to evaluate the effectiveness of serious games for climate adaptation planning processes.

What You Will Do

Your participation will involve:
- Completing two surveys individually (before and after gameplay)
- Completing a set of group level surveys during the game
- Participating in a serious game session (approximately 2 hours)
- Joining a group discussion after the game which is audio recorded

Time Commitment

Total time commitment: approximately 2.5 hours

Data Protection and Privacy

All data will be collected anonymously
- Results will be analyzed at the group level
- Your responses cannot be traced back to you individually
- Data will be securely stored in password-protected files
- Only the research team will have access to the raw data

Voluntary Participation- Your participation is entirely voluntary
- You can withdraw at any time without consequences
- You can skip any questions you prefer not to answer

Use of Results

The results will be used for:
- Publications in (academic) journals
- Improving future serious games

Participants can withdraw at any time without any consequences for their course performance/evaluation"

Contact Information

For questions about this research, please contact: Wout Sommerauer

Email: Wout.sommerauer@wur.nl

Do you have questions on the ethical permission or remarks you want to share with someone independent from the study, please contact the Wageningen University and Research - Research Ethics committee (WUR-REC).

Email: REC@wur.nl

* Vereist

1. I have read and understood the information above and agree to participate in this research. *

◯ Yes

2. What is your tracking number? *

3. Compared to before the game, has your understanding of climate adaptation challenges:

- ◯ Significantly changes
- ◯ Somewhat changed
- ◯ Slightly changed
- ◯ Not changed at all

4. Could you briefly explain your answer to question 3?

5. What sources of uncertainty do you now consider most important in climate adaptation planning (select up to 3), feel free to add your own

Selecteer maximaal 3 opties.

- ☐ Scientific model predictions
- ☐ Human behavior and societal responses
- ☐ Conflicting international interests
- ☐ A lack of technological innovation and implementation
- ☐ Political cooperation/disagreements
- ☐ Complexity and unpredictability of climate systems
- ☐ Andere

6. After the game, I expect scientific research will:

- ◯ Significantly decrease uncertainties
- ◯ Slightly decrease uncertainties
- ◯ Main current uncertainty levels
- ◯ Slightly increase uncertainties
- ◯ Significantly increase uncertainties

7. As a spatial planner, I now prefer adaptation measures that are: (Compared to pre-game)

- ◯ Robust against all climate change scenarios
- ◯ Robust against the most extreme scenario
- ◯ Tailored to the most likely scenario
- ◯ More flexible and adaptive
- ◯ No change in preference
- ◯ Andere

8. Could you explain your answer to question 7?

9. Flexibility in adaptation planning means: (Select all that apply and feel free to add your own interpretation)

- ☐ Ability to modify strategies as new information emerges
- ☐ Maintain multiple options for future action
- ☐ Minimizing long-term commitments to single approaches (not putting all eggs in one long-term basket)
- ☐ Continuous learning and adjustment
- ☐ Building resilience into planning processes
- ☐ Andere

10. I believe the largest uncertainty in projecting climate change is: (choose or add one)

- ◯ Scenario uncertainty – human behavior
- ◯ Model and statistical uncertainty
- ◯ Climate system variability
- ◯ Andere

11. Rate the importance of the following principles in adaptation planning:
(1 = Not Important, 5 = Extremely Important)

	1	2	3	4	5
Cost-effectiveness	○	○	○	○	○
Long-term sustainability	○	○	○	○	○
Community resilience	○	○	○	○	○
Ecosystem preservation	○	○	○	○	○
Equity and social justice	○	○	○	○	○

12. Has your view on uncertainty when planning for climate adaptation changed as compared to before the game? If yes, how?

13. What specific aspects of the game influenced your thinking about uncertainty?

14. Rate your agreement:
"I need absolute certainty before taking climate adaptation measures" (1 = stronly disagree, 5 = strongly agree)

1	2	3	4	5

15. The game helped me identify (additional) future uncertainties that have impact as the future unfolds
(1 = strongly disagree, 5 = strongly agree)

1	2	3	4	5

16. The game provided me with a greater understanding of the adaptation pathways concept
(1 = strongly disagree, 5 = strongly agree)

1	2	3	4	5

17. The explanation at the start of the game was easy to follow
(1 = strongly disagree, 5 = strongly agree)

1	2	3	4	5

18. The game rules were clear during the game
(1 = strongly disagree, 5 = strongly agree)

1	2	3	4	5

19. It was fun to play the serious game
(1 = strongly disagree, 5 = strongly agree)

1	2	3	4	5

20. The game should be played again next year (1 = strongly disagree, 5 = strongly agree)

1	2	3	4	5

In-game survey

In-Game Survey – quick check-in round 2

What factors influenced your decision to select these measures in this round?

Did the unexpected changes in this round impact your strategy?

a) Yes
b) No
c) Don't know/other

Could you motivate your selected answer?

On a scale of 1-10, how uncertain do you feel about the future of your adaptation strategy after this round? (1 = Not uncertain at all, 10 = Extremely uncertain)

1 2 3 4 5 6 7 8 9 10

In-Game Survey – quick check-in round 4

What factors influenced your decision to select these measures in this round?

Did the unexpected changes in this round impact your strategy?

a) Yes
b) No
c) Don't know/other

Could you motivate your selected answer?

On a scale of 1-10, how uncertain do you feel about the future of your adaptation strategy after this round? (1 = Not uncertain at all, 10 = Extremely uncertain)

1 2 3 4 5 6 7 8 9 10

In-Game Survey – quick check-in round 3

What factors influenced your decision to select these measures in this round?

Did the unexpected changes in this round impact your strategy?

a) Yes
b) No
c) Don’t know/other

Could you motivate your selected answer?

On a scale of 1-10, how uncertain do you feel about the future of your adaptation strategy after this round? (1 = Not uncertain at all, 10 = Extremely uncertain)

1 2 3 4 5 6 7 8 9 10

In-Game Survey – quick check-in round 5

What factors influenced your decision to select these measures in this round?

Did the unexpected changes in this round impact your strategy?

a) Yes
b) No
c) Don’t know/other

Could you motivate your selected answer?

On a scale of 1-10, how uncertain do you feel about the future of your adaptation strategy after this round? (1 = Not uncertain at all, 10 = Extremely uncertain)

1 2 3 4 5 6 7 8 9 10

SI2: codebook and inductive themes

Codebook for Learning and Uncertainty Analysis

Initial codes	Uncertainty type	Uncertainty object
	Epistemic	Substantive
	Ontological	Strategic
	Ambiguity	Institutional

Step 1: identified themes - Uncertainty	Predominantly linked to code
General uncertainty	Epistemic + substantive
Climate focus	Epistemic + substantive
Technical planning	Epistemic + substantive
Direct impacts	Epistemic + substantive
Environmental system response	Epistemic + substantive
Implementation reality	Ontological + strategic
Institutional	Ontological + institutional
System complexity	Ambiguity + substantive
Political dynamics	Ontological + strategic
Flexbility/Adaptation	Ontological + strategic
**Step 2: identified themes - Learning**	**Predominantly linked to code**
Systems thinking	Cognitive learning
Uncertainty Recognition	Cognitive learning
Temporal dynamics	Cognitive learning
Implementation challenges	Cognitive learning
Interconnections	Cognitive learning
Flexibility preference	Normative learning
Resilience values	Normative learning
Ethical considerations	Normative learning
Long term thinking	Normative learning
Institutional alignment	Normative learning

SI3: game guide

Game Overview

The Climate Development Pathways Game is a strategic planning simulation designed to help participants develop climate-resilient area development plans over a 50-year timeframe. Originally designed for pairs of players, the game was adapted for groups of approximately 5 people, with events displayed collectively for all participants.

Game Materials

The following materials were required per group:

- 1x situation map (A3 format)
- 1x measures game board (A3 format)
- 1x measures overview (A4 format)
- 50x characteristic cards (A4 format, cut out)
- 14x characteristic cards for vision board (when vision board printed at A0)
- 12x stop crosses per player (A4 format, cut out)
- 11x event cards (shared set for all participants) (A4 format, cut out)
- 1x purple pencil per player
- 1x green pencil per player
- Adhesive glue for each player
- PowerPoint presentation for game explanation
- PowerPoint presentation for vision changes

- Vision board poster (A0 format)
- 11 vision event cards

Game Objectives and Rules

Objective: Transform the initial area situation into a climate-resilient state within 50 years.

Winning Condition: The group achieving the highest combination of goal realization points and social acceptance points.

Game Structure: Five rounds of 10 years each (0-10, 10-20, 20-30, 30-40, 40-50 years).

Gameplay Mechanics

Initial Setup

Participants received an introduction to the game area and vision using PowerPoint presentations or printed A0 posters. Two development areas (marked with orange borders) were left undefined, allowing players to determine their own development direction (UAR/NG/WRB). Players assumed the role of civil servants tasked with developing an urgent plan within 1.5 hours, accounting for uncertainties such as climate change impacts and political changes.

Round Structure

Round 1 (Years 0-10):

- Groups selected measures to implement during the first decade
- Measures require both preparation and execution phases
- Budget constraints limited simultaneous implementation to four measures
- Measure duration was indicated by box width on the game board
- Eight measures required specification of affected area squares
- Completed measures resulted in area changes via characteristic cards placed on the situation map
- Social acceptance points were recorded for completed measures

Rounds 2-5 (Years 10-50):

- Each round began with drawing a shared event card affecting all groups
- Events modified the area vision and social acceptance points
- Groups could choose to stop ongoing measures using stop crosses
- New measures could be planned and implemented following the same mechanics as Round 1
- Area changes and point calculations continued as in Round 1
- Event-related social acceptance points were calculated and recorded

Scoring System

Goal Realization Points:

- 20 points per area square matching the final vision
- 10 points per ongoing measure that would align an area square with the vision
- Groundwater levels scored differently: Level 1 = 60 points, Level 2 = 55 points, Level 3 = 48 points, Level 4 = 40 points, Level 5 = 30 points, Level 6 = 20 points, Level 7 = 10 points, Level 8 = 0 points

Social Acceptance Points: Earned through measure implementation and event responses.

Debriefing Session

Following gameplay, a structured debriefing session examined:

- What new insights about uncertainty in climate adaptation did you gain from playing the game?
- Can you share a specific moment from your gameplay that highlighted a type of uncertainty you hadn't considered before?
- What lessons from the game could be applied to actual climate adaptation planning?

- How might you use what you learned today in your future work or studies?

SI4: Coding decision rules with anchor examples

This supplementary material provides detailed examples of how we applied the uncertainty framework by Dewulf and Biesbroek [13] to code participant responses. Each code represents the intersection of uncertainty's nature (epistemic, ontological, or ambiguity) and object (substantive, strategic, or institutional). All codes can be found in the supplementary material.

Substantive uncertainty (about outcomes, impacts, or physical changes)

Code	Definition	Pre-game example	Post-game example	Ambiguous case & resolution
**Substantive-epistemic**	Knowledge gaps about physical outcomes that could be reduced through better information	P12: "Uncertainty is what is not clear or cannot be predicted. In climate adaptation this is mainly the uncertainty in what future climate will look like."	P25: "See us government, a lot of projects stop due to there is no funding anymore"	P22: " Uncertainty relates to unknowns about what the future will look like. This can be how the economy will change, how populations will grow, how the climate system will respond. All of these are uncertain when it comes to adaptation planning and need to be taken into account" - Coded as substantive-epistemic because the framing implies these uncertainties can be managed through better knowledge and planning processes.
**Substantive-ontological**	Inherent unpredictability of outcomes regardless of knowledge	P69: "You cant predict elections, human behaviour, 1/10000 scenarios. Some things are just not predictable"	P59: "It is important that it works in practice because there will always be factors left out of models or simplified theories."	P71: "projections do not always represent future reality" - Coded as ontological because emphasis is on fundamental limits of projection itself, not on improving projection methods
**Substantive-ambiguity**	Multiple valid interpretations of what outcomes matter or what problems need solving	P26: "Uncertainty in nature and extent of climate impact, uncertainty in the effectiveness of the adaptation measures, uncertainty about the side effects both positive and negative."	P2: "It all depends on the urgency a community experiences regarding climate change impacts, the extend to which they feel they have to take up responsibility to solve the issues"	P13: "Not necessarily the same climate impacts, but rather social acceptance" - Coded as ambiguity because it questions what constitutes success (technical effectiveness vs. social acceptance), not whether we can know or predict outcomes

Strategic uncertainty (about actor behaviour, responses, or decision-making)

Code	Definition	Pre-Game example	Post-Game example	Ambiguous case & resolution
**Strategic-epistemic**	Knowledge gaps about how actors will behave that could be reduced through better understanding	P14: "That there is an uncertainty on the socio-economical and technical level. As on a social-economical level it is a challenge to understand how populations evolve and prioritise the climate change issue."	P22: "All of the other factors can be sorted out but if there is no alignment of org/gov, then we reach an institutional barrier that makes all these other factors useless"	P21: "Adaptation planning is strongly based on a current understanding of the environment, any new insights will disrupt the current planning" - Coded as strategic-epistemic because it treats actor responses to new information as a knowledge gap about how planning will be disrupted
**Strategic-ontological**	Inherent unpredictability of how actors will respond	P64: "unpredictable circumstances stemming from the combination of unpredictable sociopolitical and economic developments"	P63: "I think that the government plays a key role in implementing climate change adaptations. This can come with challenges as we have a new government every four years...Changing political arenas can result in much going back and forth"	P44: "Human behaviour and political interests change over time depending on many socio economic factors" - Coded as ontological because emphasis is on inherent changeability over time rather than our ability to understand current behaviour
**Strategic-ambiguity**	Divergent interpretations about what constitutes appropriate actor behaviour	P43: "You do not know how the future will change, due to the uncertainty in human action"	P3: "You can come up with a lot of interesting measures that could work in theory but if it doesn't work in reality we won't come a long way. That's why I think practical implementation in combination with the willingness of people for implementation and change is one of the key factors"	P26: "uncertainty in the effectiveness of the adaptation measures, uncertainty about the side effects both positive and negative" - Initially ambiguous; coded as substantive-ambiguity (not strategic) because focus is on measure effectiveness interpretation, not actor behaviour interpretation

Institutional uncertainty (about governance structures, policy continuity, or organizational systems)

Code	Definition	Clear Pre-Game Example	Clear Post-Game Example	Ambiguous Case & Resolution
**Institutional-epistemic**	Knowledge gaps about how institutions work that could be reduced through	P25: "The uncertainty is due to various stakeholders in the decision	P67: "They have the power to coordinate and agree on large scale projects"	P33: "Uncertainty is factor that come from climate change, human response, policy and

	better information	making which comes to different interest and results in different final plans are made for the futures"		economics" - Coded as institutional-epistemic because it treats policy and economics as knowable but complex factors contributing to uncertainty
**Institutional-ontological**	Inherent unpredictability of institutional and political systems	P66: "not all future events can be predicted within many sectors such as climate, politics, social attitudes, international relations, funding, etc."	P52: "Governments are in charge of the companies and people who are in a country. When not keeping their promises, the country wont either. Overall the governments should be more serious about the measures that need to be taken"	P71: "Both socio-economic and natural systems can change rapidly" - Coded as institutional-ontological (with substantive overlap) because emphasis is on rapid, unpredictable systemic change rather than knowledge gaps
**Institutional-ambiguity**	Divergent views on appropriate governance or whose responsibility adaptation is	P63: "Many of these uncertainties cannot be reduced by doing more research. Therefore we should focus on being flexible and adaptable"	P17: "If the community do not understand or want the change then I think it is gonna be really hard to implement the adaptation because people usually do not accept the changes if there is not a good enough reason for it."	P46: "you do not want to go to far because that will cost more and impact more people" - Coded as substantive-ambiguity (not institutional) because it questions the appropriate scope of intervention, not institutional roles or responsibilities

Coding principles and challenges

Multiple codes per response

Many participant responses contained multiple uncertainty types. For example, P1's pre-game response: *"Uncertainty in climate adaptation planning is crucial to acknowledge that there are multiple possible outcomes, and it comes from uncertainties related to climate systems, to modelling, and to human behaviour"* was coded as:

- **Substantive-ontological** ("multiple possible outcomes")
- **Substantive-epistemic** ("climate systems, modelling")
- **Strategic-ontological** ("human behaviour")

This resulted in 235 total coded mentions from 55 participants across pre- and post-game surveys.

Distinguishing epistemic from ontological

The most challenging distinction was between epistemic (knowledge gaps) and ontological (inherent unpredictability) uncertainty. Our decision rule:

- **Epistemic**: Language suggesting limitation is in our current understanding ("not having full understanding," "challenge to understand," "not knowing")

- **Ontological**: Language suggesting fundamental limits to prediction ("can't predict," "impossible to reduce," "will always be uncertain," "inherent unpredictability")

Distinguishing strategic from institutional

Strategic uncertainty focuses on specific actor behaviours and responses, while institutional uncertainty focuses on systemic governance structures and policy continuity. For example:

- "Communities must accept change" - Strategic (about behaviour)
- "Governments change every four years" - Institutional (about system structure)
- "Political agendas seldom agree" - Institutional (about governance dysfunction)

Ambiguity: the least common category

Ambiguity (divergent problem definitions) was relatively rare in our data, likely because:

- The game provided a shared vision map, reducing problem definition disputes
- Survey questions focused on uncertainty types rather than problem framing
- Ambiguity often manifests in stakeholder interactions rather than individual reflections

When ambiguity appeared, it typically involved questions about measure effectiveness, appropriate scope, or whose responsibility adaptation should be.

Table SI4.1: Frequency of ambiguous cases requiring interpretation

Decision Point	Frequency	Resolution approach
Epistemic vs. Ontological	Approx. 30% of cases	Prioritized language indicators: "can't know" vs. "can't predict"
Strategic vs. Institutional	Approx. 25% of cases	Focus on whether statement addressed behaviour or structure
Substantive vs. Strategic	Approx. 15% of cases	Determined primary object: outcomes or actors
Ambiguity vs. other types	Approx. 10% of cases	Required explicit problem definition divergence

Audit trail examples

To enhance transparency, we illustrate the following challenging coding decisions:

Case 1: P20 (pre-game): *"This uncertainty mostly refers to uncertainty that is for now impossible to reduce, so you can't ever be absolutely certain"*

- Initial consideration: Could be ambiguity (what does "for now" mean?)
- Final coding: Substantive-ontological
- Rationale: "Impossible to reduce" and "can't ever be absolutely certain" emphasize fundamental limits, not problem definition

Case 2: P10 (pre-game): *"Political support and economic priority are stronger than climate adaptation I believe, and therefore if there is economic gain there will be less political support."*

- Initial consideration: Strategic-epistemic (understanding priorities) or Institutional-epistemic (understanding systems)?
- Final coding: Strategic-epistemic
- Rationale: Focus on prioritization behaviour rather than institutional structure

Case 3: P34 (post-game): *"Plans can be amazing or perfectly in every single way, but every plan needs money to be actually implemented. Meaning that this has the last say over everything."*

- Initial consideration: Institutional-epistemic (resource allocation systems) or Strategic-ambiguity (what matters)?
- Final coding: Strategic-ambiguity
- Rationale: Statement questions what determines success (technical perfection vs. resources), representing problem definition ambiguity

Validation Through Triangulation

To partially validate our single-coder approach, we triangulated coded themes with unprompted content from debriefing sessions. For example:

Institutional-ontological shift identified in coding was corroborated by spontaneous debriefing statements:

- Speaker 1: "That experience of the frustration [of political change] is very visceral"
- Speaker 16: "we can't 100% prepared, but it's good to be aware of things that can happen"
- Multiple participants spontaneously mentioned "political events" as most surprising

This triangulation increases confidence that coded patterns reflect genuine participant understanding rather than coder interpretation artifacts.

Limitations of this coding approach

1. Single coder bias: Without inter-rater reliability, we cannot quantify coding consistency
2. Boundary cases: Approx. 20% of statements fell between categories, different coders might classify differently
3. Context dependence: Some statements were ambiguous without fuller response context
4. Language nuance: apprehension of the English language could have impaired responses (if applicable)

Despite these limitations, our systematic application of decision rules, focus on large-magnitude shifts, and triangulation with behavioural and debriefing data provide reasonable confidence in overall patterns, even if individual coding decisions remain interpretive.

SI5: One pager information prior to the experiment

Goal and principles of game

The serious game focuses on transforming a archetypical Dutch sandy soil area into a climate-robust environment over 50 years. Players, in groups, implement various measures across five game rounds, each representing a 10-year period. They choose and play measure cards to address environmental challenges and improve the area's resilience. As they make decisions, they track their progress with a situation map, adjusting the area's condition and earning societal acceptance points based on the measures they implement. Event cards introduce new circumstances that may require players to revise their strategies and vision for the area.

The game winner is the group with the highest score, which combines social acceptance points and points for how closely the final situation aligns with the vision. Throughout the game, players must adapt to changing conditions and manage their decisions to ensure the area becomes climate-resilient. A debriefing session at the end allows all groups to reflect on their strategies and evaluate the development pathways chosen.

Background info Dutch sandy soils

Sandy soils in the Netherlands today are primarily found in relatively elevated (compared to the western parts of the Netherlands) regions such as the Veluwe (near Wageningen) and parts of Brabant. These areas are marked by higher, dry landscapes that support forests, heathlands, towns and agricultural land. The terrain is relatively open, with sandy soils sometimes exposed, especially on the tops of the hills (see figure 1), while the lower-lying areas like the flanks of these hills and the brook valleys contain more fertile, moisture-retaining soils. These valleys support more vegetation, with small streams that add biodiversity to the landscape. Agricultural activities, particularly more intensive farming and grazing, are common in the flatter areas, though they depend on irrigation and fertilization practices due to the challenging properties of the sandy soils.

The sandy soils face significant challenges due to the changing climate and unsustainable land use practices. Sandy soils have a naturally low capacity to retain water, making them highly vulnerable to droughts. As temperatures rise and precipitation patterns shift, these regions will experience more

frequent and intense dry periods. Water shortages are expected to worsen, especially in the elevated areas, where the lack of moisture retention can lead to arid conditions. At the same time, flooding risks in the brook valleys may increase due to heavier rainfall events, leading to runoff and potential erosion. Balancing water management and land-use in these regions will be crucial to maintain agricultural productivity, ecological health, and biodiversity.

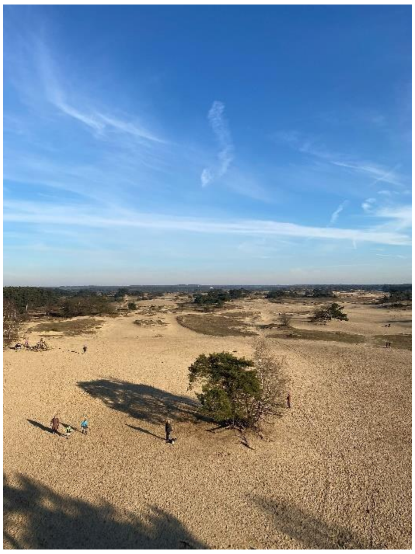

Figure SI5.1: Kootwijkerzand 02-02-2025

Other info ahead of time

Read through the sheet with potential measures to familiarize yourself in advance and potentially give you and your team an edge during the game!

SI6: significance tables and statistical testing

Table SI6.1: Thematic shifts

Construct	Pre mentions	Post mentions	Total	Expected pre	Expected post	Chi square	df	p value	phi	Effect size
**Nature: Epistemic**	26	53	79	39,5	39,5	9,23	1	0,002	0,34	Small
**Nature: Ontological**	35	75	110	55	55	14,55	1	0,000	0,36	Small-medium
**Nature: Ambiguity**	18	28	46	23	23	2,17	1	0,140	0,22	Not sig
**Object: Substantive**	35	51	86	43	43	2,98	1	0,084	0,19	Not sig
**Object: Strategic**	26	55	81	40,5	40,5	10,38	1	0,001	0,36	Small
**Object: Institutional**	18	50	68	34	34	15,06	1	0,000	0,47	Small-medium

Table SI6.2: Shifts in uncertainty

Theme	Pre n	Pre %	Post n	Post %	Expected pre	Expected post	Chi square	df	p value	phi	Effect size

**Political dynamics**	8,00	15%	31,00	56%	19,50	19,50	13,56	1,00	0,00023	0,50	Medium-large
**Flexibility/adaptation**	7,00	13%	36,00	65%	21,50	21,50	19,56	1,00	0,00001	0,60	Large
**Institutional impact**	11,00	20%	25,00	45%	18,00	18,00	5,44	1,00	0,01963	0,31	Small-medium
**System complexity**	10,00	18%	25,00	45%	17,50	17,50	6,43	1,00	0,01123	0,34	Medium
**Implementation reality**	14,00	25%	33,00	60%	23,50	23,50	7,68	1,00	0,00558	0,37	Medium
**Climate focus**	19,00	35%	8,00	15%	13,50	13,50	4,48	1,00	0,03426	0,29	Small

Table SI6.3: Group shifts - certainty

Comparison	Mean diff	SD diff	t_statistic	df	p_value	Cohen_d	Effect
Round 2→3	0,682	1,63	1,39	10	0,20	0,42	Small-med
Round 3→4	0,682	1,40	1,62	10	0,14	0,49	Medium
Round 3→5	0,63	1,54	1,36	9	0,21	0,44	Small-med

Table SI6.4: Group shifts – strategy impacted

Round	No impact	Yes/other	Total groups	Percent impact
Round 2	9	2	11	18%
Round 3	5	6	11	55%
Round 4	3	8	11	73%
Round 5	4	7	11	64%
